# Interleukin-1β Regulates Fat-Liver Crosstalk in Obesity by Auto-Paracrine Modulation of Adipose Tissue Inflammation and Expandability

**DOI:** 10.1371/journal.pone.0053626

**Published:** 2013-01-16

**Authors:** Ori Nov, Hagit Shapiro, Hilla Ovadia, Tanya Tarnovscki, Irit Dvir, Elad Shemesh, Julia Kovsan, Ilan Shelef, Yaron Carmi, Elena Voronov, Ron N. Apte, Eli Lewis, Yulia Haim, Daniel Konrad, Nava Bashan, Assaf Rudich

**Affiliations:** 1 Department of Clinical Biochemistry, Faculty of Health Sciences, Ben-Gurion University of the Negev, Beer-Sheva, Israel; 2 Chemistry and Life Sciences Program, Department of Industrial Management, Sapir Academic College, Hof Ashkelon, Israel; 3 The Goldman Medical School, Ben-Gurion University of the Negev, Beer-Sheva, Israel; 4 Department of Radiology, Soroka Academic Medical Center, Beer-Sheva, Israel; 5 Department of Microbiology and Immunology, Faculty of Health Sciences, Ben-Gurion University of the Negev, Beer-Sheva, Israel; 6 Division of Pediatric Endocrinology and Diabetology and Children Research’s Centre, University Children's Hospital and Zurich Center for Integrative Human Physiology, University of Zurich, Zurich, Switzerland; 7 The National Institute of Biotechnology in the Negev, Ben-Gurion University of the Negev, Beer-Sheva, Israel; Universidad Pablo de Olavide, Centro Andaluz de Biología del Desarrollo-CSIC, Spain

## Abstract

The inflammasome has been recently implicated in obesity-associated dys-metabolism. However, of its products, the specific role of IL-1β was clinically demonstrated to mediate only the pancreatic beta-cell demise, and in mice mainly the intra-hepatic manifestations of obesity. Yet, it remains largely unknown if IL-1β, a cytokine believed to mainly function locally, could regulate dysfunctional inter-organ crosstalk in obesity. Here we show that High-fat-fed (HFF) mice exhibited a preferential increase of IL-1β in portal compared to systemic blood. Moreover, portally-drained mesenteric fat transplantation from IL-1βKO donors resulted in lower pyruvate-glucose flux compared to mice receiving wild-type (WT) transplant. These results raised a putative endocrine function for visceral fat-derived IL-1β in regulating hepatic gluconeogenic flux. IL-1βKO mice on HFF exhibited only a minor or no increase in adipose expression of pro-inflammatory genes (including macrophage M1 markers), Mac2-positive crown-like structures and CD11b-F4/80-double-positive macrophages, all of which were markedly increased in WT-HFF mice. Further consistent with autocrine/paracrine functions of IL-1β within adipose tissue, adipose tissue macrophage lipid content was increased in WT-HFF mice, but significantly less in IL-1βKO mice. *Ex-vivo*, adipose explants co-cultured with primary hepatocytes from WT or IL-1-receptor (IL-1RI)-KO mice suggested only a minor direct effect of adipose-derived IL-1β on hepatocyte insulin resistance. Importantly, although IL-1βKOs gained weight similarly to WT-HFF, they had larger fat depots with similar degree of adipocyte hypertrophy. Furthermore, adipogenesis genes and markers (*pparg, cepba, fabp4, glut4*) that were decreased by HFF in WT, were paradoxically elevated in IL-1βKO-HFF mice. These local alterations in adipose tissue inflammation and expansion correlated with a lower liver size, less hepatic steatosis, and preserved insulin sensitivity. Collectively, we demonstrate that by promoting adipose inflammation and limiting fat tissue expandability, IL-1β supports ectopic fat accumulation in hepatocytes and adipose-tissue macrophages, contributing to impaired fat-liver crosstalk in nutritional obesity.

## Introduction

The inflammasome plays an increasingly recognized role in the pathogenesis of human diseases, including obesity and Type2 diabetes [1], [2]. Two fundamental questions remain outstanding: i. What is the specific role of various inflammasome products in obesity? Now, that cytokines other than IL-1β (e.g., IL-18, IL-33) are known to be generated by activated inflammasome, what would constitute the specific effects of IL-1β, the only cytokine of this family that can currently be clinically targeted; ii. How can systemic and/or inter-organ communication phenomena be mediated by factors mostly recognized to act locally? Can they also function directly, as endocrine mediators?

Related to obesity and type 2 diabetes, circulating levels of IL-1β were demonstrated to predict Type 2 diabetes when in conjunction with circulating IL6, suggesting a potential role for circulating IL-1β levels (though not as sole factor) [3]. At the local tissues sites, bariatric surgery-induced weight-loss was shown to diminish IL-1β expression in both subcutaneous fat and in the liver [1]. However, the most direct clinical evidence yet for IL-1β's role in the pathogenesis of type 2 diabetes has been obtained by antagonizing or neutralizing IL-1β: Treatment of Type2 diabetes patients with Anakinra (IL-1 receptor antagonist, IL-1Ra) linked this ubiquitous inflammatory cytokine mainly to the pancreatic beta-cell demise that is required for diabetes to manifest [4]. Using mouse diet-induced obesity models, targeting various components of the inflammasome, including NLRP3 [5], [6], caspase-1 [7] and ASC [7] was shown to either prevent obesity, and/or to relieve obesity-induced manifestations [5], in particular whole-body insulin resistance and hepatic steatosis [7], [8]. Yet, specifically addressing the involvement of IL-1β was more directly studied using IL-1-receptor (IL-1R) knockout mice [9]. This study demonstrated improved insulin sensitivity and prevention of IL-1-TNFα synergism in inducing adipocyte insulin resistance. Complementarily, IL-1β knockout mice were more insulin sensitive on either normal chow or high fat diet [6], [10], and both IL-1β and IL-1α knockouts were protected against hepatic steatosis induced by high fat or atherogenic diets [7], [11]. But do these represent merely effects of locally-produced IL-1β, or could arise directly or indirectly from the effect of this cytokine in other sites, thereby regulating inter-organ crosstalk?

Adipose tissue inflammation is now a well-recognized manifestation of obesity [12]. One of its major consequences is thought to be hepatic insulin resistance and steatosis, tied to adipose inflammation particularly when involving visceral fat that is drained via the portal vein, a notion known as the "portal theory" [13], [14]. This dysfunctional fat-liver crosstalk is currently best supported by several mouse models in which inflammatory mediators (Fas/CD95) and/or inflammatory signals (JNK1) were disrupted specifically in adipocytes, resulting in protection against diet-induced hepatic steatosis and insulin resistance [15], [16]. More directly, adipose tissue transplantations allowed to increase the mass of low-grade inflamed adipose tissue (secondary to the surgical procedure) independently of other effects of obesity [17]. Remarkably, only mesenteric transplantation drained via the portal vein to the liver, but not systemically-drained transplant, induced hepatic insulin resistance [17], [18]. In both human and rodent obesity adipose tissue expression of IL-1β is up-regulated, more in visceral than subcutaneous fat [1], [19], but its contribution to adipose inflammation is not clear: whereas NLRP3-KO mice do show diminished adipose macrophage infiltration [5], this was not significantly observed with IL-1RIKO [9], though macrophage inflammatory phenotype (IL-6 and TNFα secretion) was nevertheless diminished. Ex-vivo/in-vitro systems demonstrated diverse roles of IL-1β on adipocyte function, including activation of lipolysis [20], insulin resistance [21] and inhibited adipogenesis [22], [23], though the latter wasn't consistently reported [24]. Exploring the potential crosstalk with the liver, we recently demonstrated up-regulation of adipocyte expression and secretion of IL-1β in response to inflammatory stimulus [25]. Moreover, we proposed that IL-1β may constitute a mediator in the dysfunctional communication between adipocytes and hepatocytes, resulting in insulin resistance of the latter cell type [25].

In the present study we utilized a series of *ex-vivo* and *in-vivo* approaches that combine tissues from wild-type and knockout models to better define the role of IL-1β in inter-organ communication. IL-1β was found to promote adipose tissue inflammation, limit fat tissue expandability, contribute to ectopic fat accumulation and to disturbed fat-liver crosstalk.

## Results

**Figure 1:**
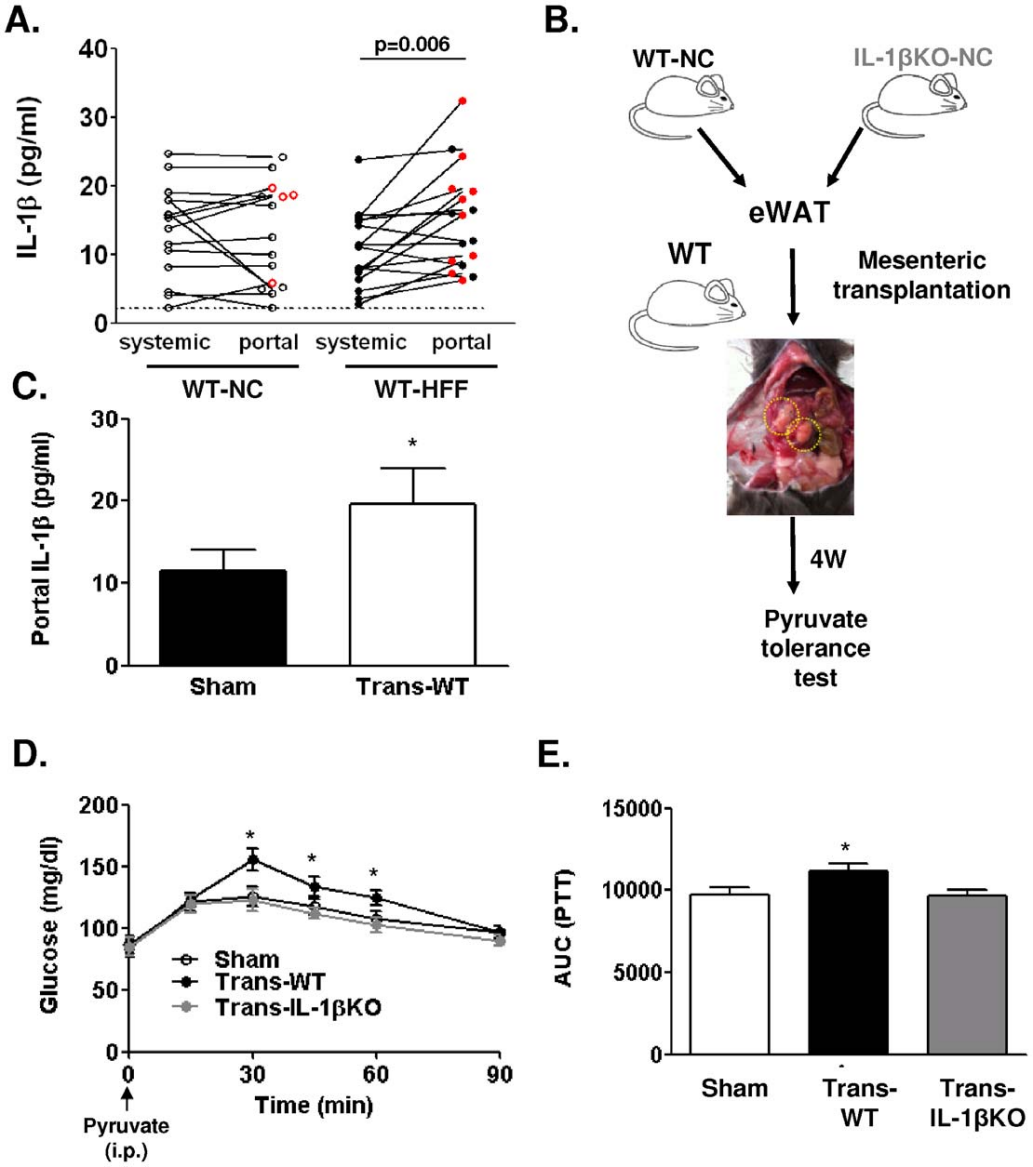
Role of adipose IL-1β in adipose-liver cross-talk as revealed by portally-drained mesenteric adipose tissue transplantation. (**A**) Serum IL-1β levels were measured in peripheral (systemic) or portal vein blood in WT mice fed normal chow (WT-NC) or high fat diet (WT-HFF). Connecting lines indicate the paired systemic-portal samples from a single mouse, n = 17–19. Red symbols represent≥20% higher IL-1β level in the portal compared to the systemic blood; (**B**) Schematic representation of the mesenteric adipose tissue transplantation experimental flow. (**C**)Portal blood levels of IL-1β were measured in sham-operated (n = 9) and in mice receiving mesenteric adipose tissue transplantation from a littermate WT mouse (Trans-WT, n = 13)*p<0.05. (**D, E**) Intra-peritoneal pyruvate tolerance test (PTT, 2 gr/Kg body weight) was performed in Sham (n = 9), Trans-WT (n = 13), and in mice receiving transplants from IL-1βKO mice (Trans-IL-1βKO, n = 7) four weeks post-transplantation. Area under the glucose levels curve (AUC) was calculated; *p<0.05 compared to Sham-operated controls.

Consistent with previous studies [5], [10], wild-type (WT) mice exhibited a time-dependent increase in adipose tissue expression of IL-1β (*IL-1b*), IL-1 converting enzyme 1 (caspase-1, *casp1*), but not IL-1α, when on high fat diet (HFF) (Fig. S1). To begin assessing a potential role for fat tissue-derived IL-1β as a direct-endocrine mediator in adipose-liver crosstalk, we measured the level of this cytokine in systemic versus portal blood. Although measured IL-1β levels were low, they were in most mice above the detection limit of the ELISA kit. HFF induced a preferential increase in IL-1β in portal blood compared to its levels in the systemic circulation in the majority of mice (Fig. 1A). To generate an *in-vivo* setting for testing the isolated effect of increased portal delivery of IL-1β, we utilized mesenteric (portally-drained) adipose tissue transplantation from WT or IL-1β-knockout (KO) mice, as recently described (Fig. 1B) [17]. Transplanted WT mice indeed had elevated portal vein levels of IL-1β (Fig. 1C), consistent with the procedure-associated low-grade inflammation of the transplant [17]. To specifically address the role of the fat-transplant-related IL-1β on liver function, we assessed the pyruvate-glucose flux in mice receiving transplant from either WT or IL-1βKO mice. Compared to sham-operated controls, mice receiving a transplant from WT, but not IL-1βKO mouse, had a higher rise in blood glucose levels during pyruvate tolerance test (PTT, Fig. 1D, E). This finding suggests that the absence of IL-1β in the transplant prevented the augmented conversion of pyruvate to glucose induced by increased mass of portally-drained adipose tissue.

**Figure 2:**
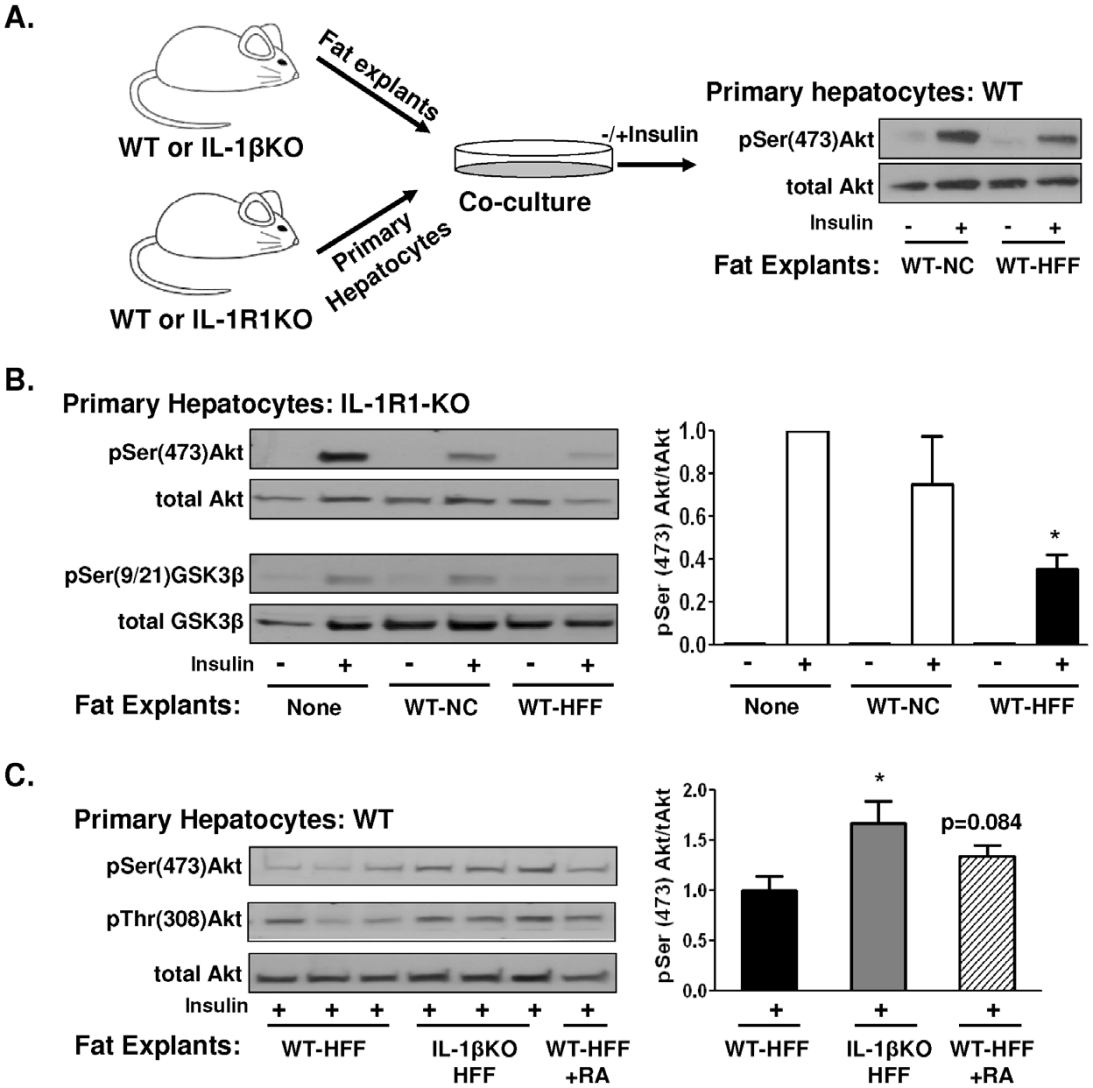
Role of adipose IL-1β in hepatocyte insulin resistance as revealed by co-culture approach. (**A**) Schematic representation of the fat explants – primary hepatocyte co-culture experimental design. (**B**) Insulin-stimulated Akt and GSK3 phosphorylation in primary hepatocytes from IL-1RIKO liver co-cultured or not with fat explants from WT-NC or WT-HFF and densitometry analysis of 2–5 mice per group. *p = 0.05 compared to incubation with fat explants from WT-HFF mice. (**C**) Insulin-stimulated Akt phosphorylation in primary hepatocytes from WT mice co-cultured with fat explants from WT-HFF, IL-1βKO-HFF, or WT-HFF in the presence of IL-1 receptor antagonist (WT-HFF+RA). The right graph depicts densitometry analysis of 7–9 mice per group. *p<0.05 compared to the signal obtained from primary hepatocytes incubated with fat explants from WT-HFF mice.

To determine if adipose-derived IL-1β can directly regulate fat-liver communication or rather act locally to alter adipose tissue adaptation in obesity, we utilized co-culture of primary hepatocytes with adipose tissue explants (Fig. 2A). Adipose tissue explants from WT-NC mice somewhat attenuated insulin-stimulated Akt though not GSK3 phosphorylation in primary hepatocytes from IL-1RI (i.e., isolated from IL-1R1KO mice, Fig. 2B, C). In addition, a significant, near-complete diminution of hepatocyte insulin responsiveness was induced by adipose explants from WT-HFF mice, suggesting an effect independent of hepatocyte IL-1RI (Fig. 2B, C). Furthermore, primary hepatocytes from WT mice co-cultured with adipose tissue explants from IL-1βKO-HFF mice responded better to insulin stimulation than the same cells incubated with fat explants from WT-HFF mice. However, blocking the direct effect of IL-1β with IL-1 receptor antagonist (IL-1Ra) only partly and with marginal statistical significance corrected insulin responsiveness of WT hepatocytes co-cultured with WT-HFF explants. Collectively these results demonstrate that diet-induced obesity in the absence of IL-1β alters adipose tissue in a manner less detrimental to adipose-liver crosstalk. Yet, in WT conditions when IL-1β is present, it does not seem to directly mediate the disturbed fat-liver crosstalk induced by obesity. Rather, adipose-derived IL-1β seems to act primarily locally to regulate adipose tissue adaptation to obesity, consequently resulting in impaired fat-liver crosstalk.

**Figure 3:**
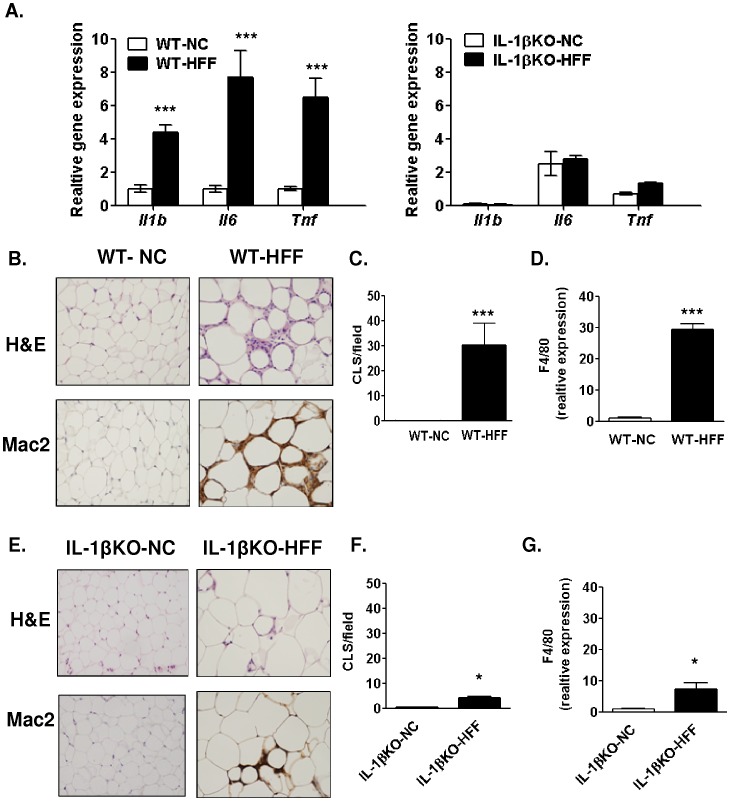
High fat feeding induces only a minor adipose tissue macrophage infiltration in IL-1βKO mice. (**A**) Quantitative real-time PCR analysis of adipose tissue (epididymal fat pad) of *IL-1b, il6* and *tnf* (normalized to *tbp*, *18S* and *36b4*). n≥3 per group. (**B–D**) Representative X20 light microscopy images of adipose tissue stained with H&E or with anti-Mac2 antibody. The mean±SEM number of crown like structures (CLS) per X10 microscopic field was counted as described in Materials and Methods. mRNA levels of *f4/80*, a macrophage marker, was assessed by quantitative real-time PCR. (**E–G**) Similar analysis as described above (B–D), but for IL-1βKO-NC and IL-1βKO-HFF mice. n = 3–6 animals per group were included in the analysis. *p<0.05 compared to IL-1βKO-NC; ***p<0.001 compared to WT-NC.

To begin addressing the local role of IL-1β in adipose adaptation to diet-induced obesity, we tested the effect of HFF on adipose tissue expression of pro-inflammatory cytokines in IL-1βKO mice (Fig. 3A). As expected, *IL-1b* mRNA levels were non-detectable in IL-1βKO mice on either diet. Importantly, in the absence of IL-1β, HFF induced only a 1.1-and 1.8–fold increase in *il6* and *tnfa* mRNA, much less than in WT mice. Histological sections of adipose tissue stained with haematoxylin-eosin (H&E) or immunostained with anti-Mac2 revealed that HFF induced a significant infiltration of macrophages in crown-like structures (CLS, inflammatory cells surrounding adipocytes), whereas this was less evident in IL-1βKO mice (Fig. 3B, C, E, F). Complementarily, *f4/80* mRNA, a marker of macrophage infiltration into the tissue measured by quantitative real-time PCR, revealed only a 7.4-fold induction by HFF in the IL-1βKO, much less than in- WT mice (31.7-fold) (Fig. 3D,G).

**Figure 4:**
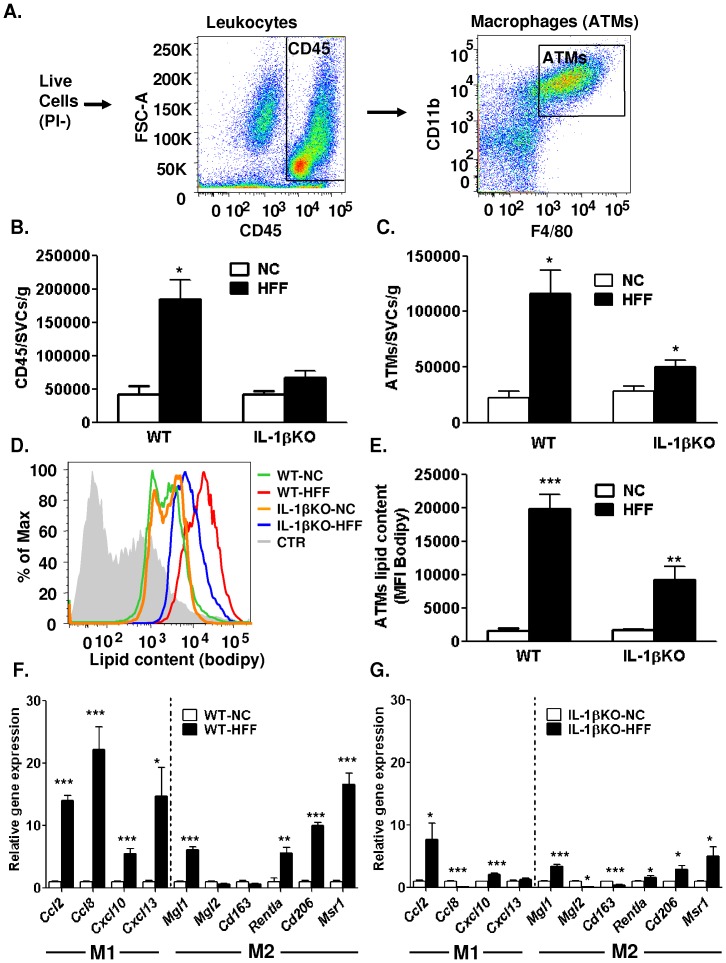
Role of IL-1β in adipose tissue macrophage recruitment, ATM lipid content, and adipose inflammatory profile in dietary obesity. (**A**) FACS plots and gating of the stromal-vascular cells (SVCs) to detect adipose tissue macrophages (ATMs). Leucocytes (**B**), ATMs (**C**) in adipose tissue of WT-NC (n = 4), WT-HFF (n = 11), IL-1βKO-NC (n = 3) and IL-1βKO-HFF (n = 7). (**D**) Histogram of lipid content (determined with Bodipy) in representative mice of the 4 mouse groups (**E**)**.** Quantitative real-time PCR analysis of M1 or M2- genes in epididymal adipose tissue of (**F**) WT-NC, WT-HFF (n = 4, 11, respectively), and (**G**) IL-1βKO-NC and IL-1βKO-HFF (n = 3 and 7, respectively). The expression of each transcript was normalized to *tbp*, *18S* and *36b4* mRNA/rRNA, and a value of 1 was assigned to the normal chow group (NC) of each strain. *p<0.05, compared to NC; **p<0.01 compared to NC ***p<0.001.

We further characterized how the lack of IL-1β altered the non-adipocyte cellular component of adipose tissue in response to HFF using FACS analyses. Following isolation of the SVF and exclusion of dead (propidium iodide–positive) cells, SVC were gated for CD45 to detect leukocytes, and in this fraction macrophages were identified based on double-positive staining of F4/80 and CD11b (Fig. 4A). Consistent with other reports [26], obesity greatly increased the number of CD45-positive cells and adipose tissue macrophages (ATMs, Fig. 4B, C, respectively). However, IL-1βKO-HFF mice exhibited only a non-significant increase in total number of leukocytes, and only a small increase in the number of ATMs. ATMs may accumulate triglycerides by engulfing lipid droplet remains of dead adipocytes [19], and/or by re-esterifying free fatty acids released from adipocytes [27]. We therefore used the fluorescent neutral lipid stain Bodipy to assess ATM lipid content (Fig. 4D). In WT mice HFF increased 12.1-fold the mean lipid fluorescence in ATMs compared to WT-NC. IL-1βKO-HFF however exhibited a significant attenuation of HFF-induced increase in the mean ATMs' lipid content (Fig. 4E). Complementarily, cultured J774.1 mouse macrophage cells incubated in-vitro with free fatty acids or aggregated LDL revealed that adding IL-1β to the culture medium enhanced lipid accumulation (Fig. S2A, B), consistent with a previous report demonstrating such effect with both IL-1β and TNFα [28].

The findings above offered a putative effect of locally-produced IL-1β along with adipose tissue microenvironment on ATMs. Further along this line, we assessed how in the absence of IL-1β HFF altered the inflammatory profile of adipose tissue and ATMs by measuring the mRNA levels of several M1/M2 genes ("classically-activated"/"alternatively-activated" macrophages) (Fig. 4F,G) [29]. Consistent with other reports in mice [30] and in humans [31], obese mice showed a significant increase in M1 and in some of the M2 genes. In IL-1βKO mice this obesity induced change in the M1 and M2 genes was markedly less pronounced. Collectively, the adipose tissue response to HFF in IL-1βKO mice uncovers a major role for IL-1β in the development of adipose tissue inflammation in response to diet-induced obesity.

**Figure 5:**
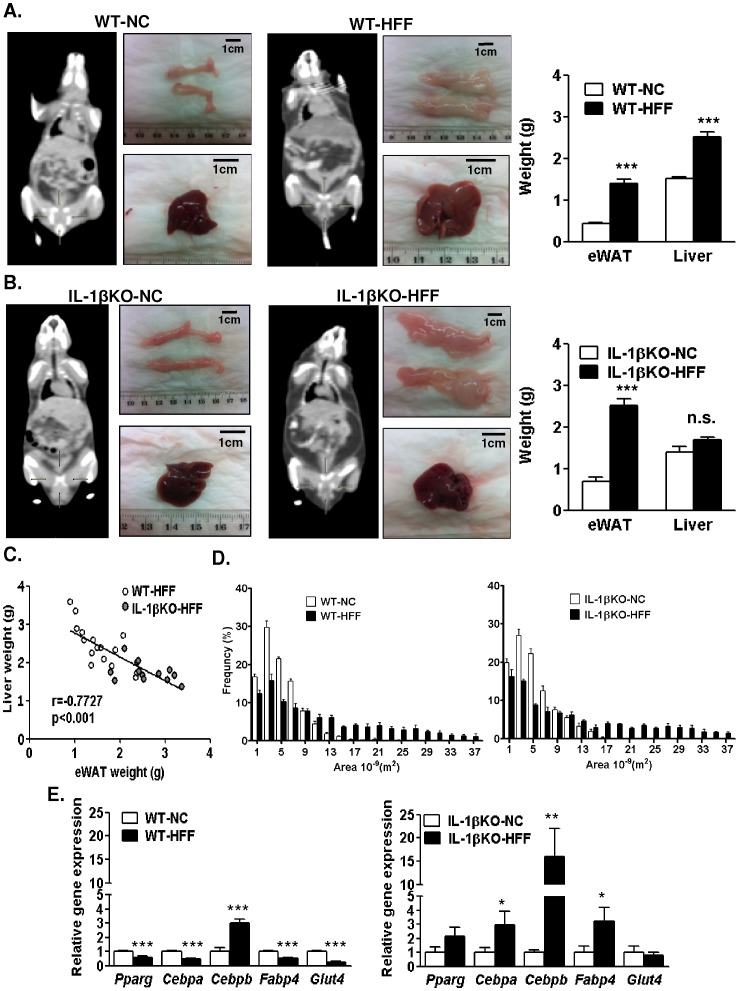
IL-1β impact on liver and adipose tissue mass and adipose tissue expandability. (**A**) Representative Computed Tomography (CT) scans (mid-coronal sections) of WT-NC and WT-HFF mice, and excised epididymal white adipose tissue (eWAT) and livers, and the mean±SEM of their weights. (**B**) Similar to A, but for IL-1βKO mice. ***p<0.001 compared to NC. (**C**) Spearman correlation between epididymal fat pads' weight and liver weight in HFF mice. (**D**) Adipocyte size distribution in WT and IL-1βKO mice, quantified as described in Methods. n = 3–6 mice per group. (**E**) Quantitative real-time PCR analysis of the indicated genes in epididymal adipose tissue in WT and IL-1βKO mice, respectively. n = 3–6 per group. *p<0.05 compared to IL-1βKO-NC ***p<0.0001 compared to WT-NC.

Adipocyte hypertrophy and associated cell death may be major drivers of adipose tissue inflammation in obesity. Conversely, cellular hyperplasia may support increased adipose tissue expandability that prevents "ectopic" triglyceride storage in non-adipose tissues like the liver. We therefore tested if the attenuated HFF-induced adipose tissue inflammation in IL-1βKO-HFF mice was associated with altered adipose tissue expandability. Body composition analysis using computed tomography (CT) revealed that HFF caused expansion of adipose tissue compartments in WT mice. After 16w on HFF, both epididymal fat and liver weights were significantly increased compared to WT-NC (Fig. 5A). Remarkably, although IL-1βKO gained similar total body weight compared to WT mice when on HFF (Fig. S4), adipose tissues accumulation appeared to exceed that which was observed in WT-HFF mice in both the intra-abdominal cavity and in subcutaneous depots (Fig. 5B). Yet, no similar increase in liver size was observed, in contrast to WT mice (Fig. 5A, B). This corresponded histologically to the extent of lipid accumulation, with 56.1±6.9% versus 27.5±5.5% of the sections area being steatotic in WT-HFF versus IL-1βKO-HFF, respectively (Fig. S3). Interestingly, in HFF mice a higher fat mass correlated with lower liver weight (Fig. 5C), supporting the proposition that in the absence of IL-1β greater expansion of adipose tissue protected against ectopic fat accumulation in the liver.

To gain further insight on the mechanisms for increased adipose tissue expandability in IL-1βKO-HFF mice we assessed adipocyte size distribution. Using a semi-automated quantitative analysis of histological sections revealed that overall HFF induced a similar shift towards larger adipocytes in IL-1βKO and WT mice (Fig. 5D). This suggests that the increased adipose tissue mass of IL-1βKO-HFF compared to WT-HFF could not be attributed to greater diet-induced adipocyte hypertrophy, and therefore largely relied on an increase in adipocyte cell number. Consistently, whereas in WT mice HFF induced a decrease in early (*pparg, cebpa*) and late (*fabp4, glut4*) adipogenic markers, as previously reported [32], [33], IL-1βKO mice showed significant increase in *pparg, cebpa and fapb4* and no change in *glut4* (Fig. 5E). This unique increase in adipogenic genes in response to HFF could support augmented adipose tissue expansion in the absence of IL-1β.

## Discussion

The fundamental role of IL-1β in inflammatory cascades is well established, as is now the recognition of adipose tissue inflammation in obesity-associated-morbidities. Yet, whether and how IL-1β regulates adipose inflammation and fat-liver crosstalk in obesity is poorly defined. The present study reveals that: i. IL-1β regulates lipid storage capacity in adipose tissue, in ATMs, and as previously shown, in the liver [11]. In its absence, adipose tissue expandability increases in response to excess calories, correlating with lower liver weight and less (ectopic) lipid storage. ii. IL-1β is a major promoter of adipose tissue inflammation in obesity. iii. IL-1β regulates adipose-liver crosstalk, mainly having local effects in both tissues. IL-1β-mediated autocrine/paracrine actions in adipose tissue that promote local inflammation and limit expandability generate a dysfunctional fat-liver communication that could contribute to liver steatosis and insulin resistance.

### Regulation of TG Storage in Obesity

Previous studies have demonstrated local effects of IL-1β on either adipocytes or hepatocytes, both of which can contribute to hepatic lipid accumulation in obesity: In cultured adipocytes IL-1β was shown to induce lipolysis by down-regulating PPARγ [20]. Such an effect in an *in-vivo* setting would result in increased free fatty acid delivery to the liver. Complementarily, IL-1β may directly limit hepatocyte fat oxidation by inhibiting PPARα [8]. Consistent with these studies, our study shows that IL-1β deficiency results in reduced liver steatosis and reduced insulin resistance. However in addition, our findings suggest that IL-1β may also limit adipose tissue expandability: in its absence a marked increase in whole-body fat mass was observed. Intriguingly, adipocytes of IL-1βKO-HFF showed a similar cell-size distribution to that seen in WT-HFF, implying that the larger fat depot mass is the result of larger total number of adipocytes (i.e., hyperplasia). IL-1β was previously shown to inhibit adipogenesis [22], [23], and here we show that whereas in WT mice HFF decreases expression of genes involved in adipogenesis, the opposite response to HFF is induced in IL-1βKO mice, revealing an IL-1β-mediated blockade on adipose tissue expandability.

Limited adipose tissue expandability, particularly in subcutaneous fat, has been suggested to exert a causative role in obesity-associated morbidity (the "ectopic fat/lipid overflow" theory [34]). According to this view, adipose tissue is the metabolically safe site for excessive lipid storage, hence when storage capacity is exhausted, lipid overflows to non-adipose tissues causing ectopic fat accumulation and in turn, metabolic dysfunction [35]. Hypertrophied adipocyte cell death could trigger adipose inflammation, and pro-inflammatory cytokines (including IL-1β) can inhibit key adipogenesis genes like *pparg*. Thus, it is likely that IL-1β participates in either hypertrophic adipocyte cell death that launches the inflammatory cascade, and/or directly in limiting adipose hyperplastic response and expandability. Jointly, these contribute to ectopic fat accumulation, impaired adipose-liver crosstalk and obesity-associated morbidity.

### IL-1β Regulation of Adipose Tissue Inflammation in Obesity

What is IL-1β's role in adipose tissue inflammation in obesity? In the absence of IL-1β total adipose tissue leukocyte and macrophage counts were lower under HFF, and obesity-associated increase in expression of M1 (classically-activated) macrophage markers seemed less pronounced (*ccl2, cxcl10, cxcl13*) or completely absent (*ccl8*). HFF-induced changes in M2 (alternatively-activated) macrophage markers were also lower in IL-1βKO mice. This suggests that a major role of IL-1β in adipose tissue inflammation is in the recruitment of macrophages to adipose tissue, although a more subtle regulation of specific macrophage inflammatory signature markers is plausible. These findings are consistent with observations in NLRP3-KO mice [5], [6]. Although the overall conclusion (i.e., of a key role for the IL-1β/IL-1R in obesity-associated adipose tissue inflammation) is well consistent with the findings in IL-1RIKO mice [9], the inconsistencies are also worth noting given that both models target the exact biological system. In IL-1RIKO mice ATMs were functionally less inflammatory, but their numbers (both total and M1 macrophages defined by being CD11c+) were not changed compared to WT. The reasons for this apparent discrepancy are unknown, and could result from differences in experimental conditions (different diets were used in both studies), from different compensatory changes induced by knocking out each of the two genes, and/or represent true biological differences when targeting the cytokine versus its receptor. For example, absence of the receptor, even without inducing compensatory up-regulation of receptors for other pro-inflammatory cytokines, could induce changes in abundance/availability of intracellular receptor partners that are common between IL-1RI and TLR's signaling. Interestingly, in our study lack of IL-1RI in primary hepatocyte did not prevent insulin resistance induce by adipose tissue explants from WT-HFF mice, whereas the absence of IL-1β in the adipose tissue fragments did suggest involvement of IL-1β in adipose-hepatocyte crosstalk. Moreover, in the leptin/leptin receptor mice models, hepatic steatosis was more severe in ob/ob mice than in db/db mice [36] – consist with a somewhat milder phenotype when deleting the receptor versus its ligand. We propose that closer investigation into these differences may reveal new insights into the specific input of the "IL-1 system" in obesity.

Our findings also provide *in vivo* evidence for a role for IL-1β in promoting triglyceride accumulation in ATMs. Such activity is reminiscent of *in vitro* studies using cultured (non adipose-derived) macrophages [37] and when the effect of IL-1β was tested on cholesterylester-laden macrophages [38]. Thus, our results suggest that IL-1β is a regulator of adipose inflammation by promoting leukocyte and macrophage recruitment, and interestingly, by enhancing macrophage lipid accumulation.

### The Role of IL-1β in Adipose-liver Crosstalk

Using cultured cell lines we have previously proposed an endocrine role for IL-1β in adipocyte-hepatocyte communication in response to adipocyte inflammation [25]. Here we estimated the role of IL-1β in adipose-liver crosstalk by co-culturing fat explants with primary hepatocytes. Insulin responsiveness was higher in hepatocytes co-cultured with adipose explants from IL-1βKO-HFF than WT-HFF mice. A direct role of IL-1β as an endocrine mediator in adipose-liver crosstalk could be supported by demonstrating a preferential increase in portal (over systemic) IL-1β levels in response to HFF. Yet, in the fat explant/primary-hepatocyte system, antagonizing IL-1β with IL-1Ra only partially, and with marginal statistical significance, prevented the insulin resistance observed in liver cells. Furthermore, hepatocytes from IL-1RIKO mice were not protected against the insulin resistance induced by co-culturing them with adipose tissue explants from HFF mice. Thus, only a minor, if any, direct endocrine function of adipose tissue-derived IL-1β on hepatocytes may be operational. Rather, it is most likely that IL-1β's role in regulating the endocrine function of adipose tissue is mediated by its own contribution to adipose tissue inflammation via autocrine/paracrine actions discussed above. The mesenteric transplantation of adipose tissue from IL-1βKO mice may suggest a unique role for IL-1β specifically in the adipocytes in this local autocrine-paracrine function, as host-derived infiltrating cells into the transplant will have had IL-1β. Indeed, compared to transplantation from a WT donor, mice exhibited a lower pyruvate to glucose flux. Jointly, it appears that IL-1β may mainly exert autocrine/paracrine effects in adipose tissue that consequently deteriorate metabolic adipose-liver crosstalk in obesity.

### A Putative Pathway for Adipose IL-1β in the Pathogenesis of Obesity-associated Morbidity

Our findings along with current literature may suggest the following patho-physiological pathway: Diet induced obesity induces adipose tissue inflammation that includes IL-1β up-regulation, which contributes to the recruitment of adipose tissue macrophages and to the induction of additional pro-inflammatory cytokines. These in turn act to limit hyperplastic adipose tissue expansion, thereby promoting ectopic fat accumulation both within and outside adipose tissue (ATMs and hepatocytes, respectively). Ectopic fat accumulation and adipose tissue–derived inflammatory mediators, then contribute to hepatic steatosis, hepatic insulin resistance, and augmented pyruvate-glucose flux.

## Materials and Methods

### Animals and Treatments

The study was approved in advance by Ben-Gurion-University Institutional Animal Care and Use Committee, and was conducted according to the Israeli Animal Welfare Act following the Guide for care and Use of Laboratory Animals (National-Research Council, 1996). Male wild-type (WT) C57Bl/6 mice were purchased from Harlan Laboratories (Rehovot, Israel). IL-1β homozygote knock-out (IL-1βKO) mice on C57Bl/6 background were generated and used [39], [40] as described [41]. Animals were housed, in the preclinical facility, 2–3 per cage in individually ventilated cages, which were changed aseptically at least every week in a class 2A1 biosafety cabinet. Animals were housed in 12∶12 light:dark cycle at 20–24°C and 30–70% relative humidity and had free access to reverse osmosis filtered water. Mice were allowed free access to autoclaved normal rodent chow (NC, 11% calories from fat, 65%- carbohydrates and 24%– protein, Altromin, Lage, Germany). For diet-induced obesity studies, high fat feeding (HFF) was initiated in parallel in IL-1βKO or WT mice at age 6–7 weeks using diet consisting of 58.7% calories from fat, 25.5%- carbohydrate, and 15%- protein (Research Diets, New Brunswick, NJ, D12492) as described in [42]. Due to occasional sporadic cases of dental abscesses in IL-1βKO mice, all mice (WT and KO) were given 1.25 ml 5% Enrofloxacin (Bayer Healthcare, Leverkusen, Germany) in drinking water every second week, with no effect of antibiotics on weight gain, development of glucose intolerance or insulin resistance when on HFF. Blood was drawn from the tail (100 µl) for fasting insulin and glucose measurements. At the end of the experiment mice were killed with CO_2_ or isoflurane, and tissues were obtained and processed as detailed in following sections. Both C57Bl/6 strains showed a similar weight gain on HFF compared to mice fed NC (Fig. S4A, B). Yet, while WT-HFF mice progressively developed impaired insulin tolerance and insulin resistance (by ITT and HOMA-IR, respectively, (Fig. S4C–F, Fig. S5A–C)), IL-1βKO-HFF were protected against these endocrine/metabolic effects of diet-induced obesity.

### IL-1β Measurements

The levels were performed in serum by ELISA (Quantikine, R&D Systems, cat-MLB00C), following the manufacturer's instructions with the following adjustments: orbital shaking (500 rpm, 2 h) during the Assay-Diluent stage and the following IL-1β conjugate step. This shaking was essential to decrease the lower detection limit of the kit to 2.35 pg/ml (lowest concentration in the standard curve, with a coefficient of variance (CV) of 14.9%).

### Portally-drained Mesenteric Fat Transplantation

The transplantation was performed at 8 weeks of age on WT mice as described [17]. Briefly, both epididymal fat pads were removed from WT or IL-1βKO donor mice, rinsed with 0.9% saline and stitched to a recipient's mesenterium using Assu-cryl 6.0 (Assut-Medical, Corgemont, Switzerland). Sham-operated control mice undergone identical procedures and an artificial suture was performed. The peritoneum was sealed by Assu-cryl 5.0. Surgical skin wounds were closed by Reflex skin closure system (Stoelting, Wood Dale, IL) and 7 mm clips (CellPoint Scientific, Gaithersburg, MD). Mice were given 2.5 ml 5%Enrofloxacin and 1.25 g Dipyrone syrup (Vitamed, Binyamina, Israel) in drinking water for 3 days. After 4 weeks, during which weight gain was identical between transplanted and sham-operated mice, mice were used for metabolic assessment as described below.

### Primary Mouse Hepatocytes and Co-culture with Adipose Tissue Explants

Cell isolation was performed as described [43]. Briefly, livers from WT mice or IL-1RIKO mice (purchased from The Jackson Laboratory (Bar Harbor, ME)) were perfused via the inferior vena-cava with Krebs Ringer buffer supplemented with 0.1 mM EGTA, followed by the same solution with 0.5 mg/ml collagenase Type1 (Worthington Biochemical Corporation, Lakewood, NJ). Hepatocytes were collected, centrifuged, washed and plated (0.25×10^6^ cells/well in 6 well collagenI-coated plates) with DMEM containing 1 g/L glucose and 10% FBS followed by DMEM/F12 (1∶1) media for 24 hours. Cells were co-cultured for 24 h with ∼100 mg epididymal fat fragments from WT or IL-1βKO mice on HFF after over-night relaxation. IL-1Ra (100 ng/ml) was added where indicated to the medium. At the end of the co-culture period explants were removed, and hepatocytes stimulated with 100 nM insulin for 7 min. Cells were then washed and lysates prepared for western blot analysis.

### FACS Analysis of Adipose Tissue

Epididymal fat pads were excised and minced in 10 ml of DMEM containing 4.5 mM glucose (without phenol-red), 2 mM HEPES pH7.4, and 2%BSA. CollagenaseII (Sigma C6885; 1 mg/ml) was added, and minced tissues were incubated at 37°C for 20 min with shaking. After removing large particles using 250 µm sieves, 10 mM EDTA was added, followed by 2 centrifugations (500 g, 5 min, 4°C) to separate ﬂoating adipocytes from the stromal-vascular fraction (SVF) pellets. Following washing, cells were re-suspended in 300 µl of staining buffer (PBS containing 2%FBS) containing FcBlock (BD-Biosciences, Franklin Lakes, NJ) and stained with the following conjugated antibodies (all on ice, 10 min in the dark): CD45-APC, F4/80-PE-Cy7 (both from E-Bioscience, San-Diego, CA) and CD11b-APC-Cy7 (BD-Pharmingen, San-Diego, CA). Thereafter, cells were washed and pellets were stained for 20 min with Bodipy 493/503 (3 µg/ml Bodipy for 5×10^6^ cells, Invitrogen). Stained samples were further washed and filtered using 100 µm mesh. Propidium Iodide (0.2 µg/ml, Sigma) was added to all samples. Stained samples were analyzed by FACS (Canto, BD-Biosciences, Franklin Lakes, NJ).

### RNA Extraction and Quantitative RT-PCR

Total RNA from fat pads was extracted with the RNeasy lipid tissue mini kit (Qiagen, Germantown, MD) and analyzed with Nanodrop©. RNA (200 ng) was reverse-transcribed with High-Capacity cDNA Reverse Transcriptase Kit (Applied Biosystems, Foster City, CA). Taqman system (Applied-Biosystems, Foster City, CA) was used for real-time PCR amplification. Relative gene expression was obtained after normalization using the formula 2-ΔΔCT, using specific primers (Table S1).

### Cell Lysates and Western Blot Analysis

Protein lysates (in RIPA buffer) and Western blot analysis were performed as previously described [25], and bands quantified as using ImageGauge software (Ver. 4.0, Fuji Photo-Film, Tokyo, Japan) [44]. In each experiment an insulin-stimulated control received a value of 1 arbitrary unit, and all samples intensities were expressed as that value. Antibodies used were: anti-pro-IL-1β antibody (Abcam, San-Francisco, CA); anti–pSer473-PKB/Akt antibody, anti–pSer308-PKB/Akt antibody, anti-PKB/Akt antibody, anti-pSer9/21-GSK3β antibody, and anti-GSK3β antibody were from Cell Signaling (Beverly, MA), anti-β-actin and anti-β-tubulin were from Sigma (St. Louis, MO).

### Histology

Haematoxylin-eosin and immunohistological staining were performed in sections of paraffin-embedded epididymal fat and livers exactly as described previously [45], [46]. The number of crown like structures (CLS) was determined in 6 different ×10 fields from 3–6 mice per group. The percentage of steatosis in liver histological sections was estimated in 6 different X10 fields from 3–6 mice from H&E stained sections by two independent observers blind to the treatment group. All pictures were taken using an Olympus DP70 microscope. Oil red O staining of liver sections was performed as described [47].

### Fat Cell Size Estimation

The estimation was based on X10 magnification of histological sections images (SPOT digital camera, Diagnostic Instruments, Sterling Heights, MI). Images were converted into a binary format with ImageJ (1.45S Wayne Rasband, NIH, USA) and compared with the original images to ensure an accurate conversion. Cross-sectional areas<350 µm^2^ was considered as technical artifact and ignored. 50–100 cells/group were measured in 5–7 different X10 fields from 3–6 mice.

### Computed Tomography (CT) Scanning

Scans were performed in mice anesthetized with Ketamine and Xylasine (i.p., 100 and 10 mg/kg body weight, respectively) in saline. Images were acquired on Philips Mx8000 IDT 16.

### Pyruvate Tolerance Tests (PTT)

PTT was performed by i.p injection of 2 g/kg body weight pyruvate in saline after over night fast, and tail blood measurements every 15 minutes using a glucometer (Abbott, Alameda, CA).

### Statistical Analysis

Data are expressed as the mean±S.E.M. statistically significant differences between two groups were evaluated using paired or non-paired Student's t–test as required (GraphPad software). Correlation between liver weight and eWAT weight was assessed using Spearman correlation. p<0.05 was considered statistically significance.

## Supporting Information

Figure S1 **Increased adipose tissue expression of IL-1β in diet induced obesity.** (A) Quantitative real-time PCR analysis of interleukin 1β (*IL-1b*), interleukin 1α (*IL-1a*) and caspase1 (*casp1*) in epididymal adipose tissue of C57/Bl6 wild-type (WT) mice during high fat feeding (HFF). Values are adjusted to 18S rRNA, and presented relative to age-matched littermates on normal chow diet (WT-NC). n = 5–12 per group/time-point; * p<0.05 compared to WT-NC at the same time point. (B) Representative western blot and densitometry analysis of pro-IL-1β in epididymal fat of 16 weeks high fat fed (HFF) or normal chow (NC) wild-type (WT) mice. A value of one was assigned to the mean pro-IL-1β to β-tubulin ratio in WT-NC. n = 5 in each group; *p<0.05.
(PPT)

Figure S2 **IL-1β contributes to macrophage lipid accumulation. (A)** J774.1 mouse macrophage cell line was incubated with aggregated LDL or with 0.5 mM oleic acid in the absence or presence of the indicated IL-1β concentrations. After 18 h cells were stained with oil red o to stain neutral lipids, and light microscopy images were taken. **(B)** Cells from 5 independent experiments were dissolved with DMSO and absorbance was determined using a microplate reader assigning an arbitrary value of 1 to control cells incubated in the absence of either lipids or IL-1. * p<0.05 compared to control.
(PPT)

Figure S3 **High fat diet induced hepatic steatosis is enhanced by IL-1β.** Histological sections of livers of WT-NC, WT-HFF, IL-1βKO-NC and IL-1βKO-HFF stained for haematoxylin and eosin (H&E). WT-HFF and IL-1βKO-HFF were also stained for the neutral lipid stain Oil red O as detailed in methods. Shown are representative images of X20 light microscopy fields for H&E and oil red O.
(PPT)

Figure S4 **IL-1β contributes to the development of fasting hyperinsulinemia in response to high-fat diet. (A–B)** Body weight dynamics in WT-NC, WT-HFF (n = 8–16) and in IL-1β knockout mice on normal chow (IL-1βKO-NC) or high fat diet (IL-1βKO-HFF) (n = 6–13) as a function of weeks of dietary intervention. WT-HFF were not significantly different from IL-1βKO-HFF in any of the time points, but differed significantly (p<0.01) from WT-NC or from IL-1βKO-NC from week 3 respectively. The same groups were assessed for fasting insulin **(C–D)** and homeostasis model assessment insulin resistance (HOMA-IR)**(E–F).** * p<0.05 compared to WT-NC.
(PPT)

Figure S5 **IL-1β contributes to whole-body insulin resistance in response to diet induced obesity. (A–B)** Insulin tolerance test (ITT, 0.2 U/Kg body weight after 3 h fasting) after 12 weeks of HFF or normal chow, and **(C)** calculated area under the curve (AUC). * p<0.05 compared to WT-NC.
(PPT)

Table S1 **List of Taq-man primers for quantitative real-time PCR.**
(PPT)

## References

[1] Moschen AR, Molnar C, Enrich B, Geiger S, Ebenbichler CF, et al (2011) Adipose and liver expression of interleukin (IL)-1 family members in morbid obesity and effects of weight loss. Mol Med 17: 840–845. 21394384 10.2119/molmed.2010.00108 PMC3146615

[2] Stienstra R, Tack CJ, Kanneganti TD, Joosten LA, Netea MG (2012) The inflammasome puts obesity in the danger zone. Cell Metab 15: 10–18. 22225872 10.1016/j.cmet.2011.10.011

[3] Spranger J, Kroke A, Mohlig M, Hoffmann K, Bergmann MM, et al (2003) Inflammatory cytokines and the risk to develop type 2 diabetes: results of the prospective population-based European Prospective Investigation into Cancer and Nutrition (EPIC)-Potsdam Study. Diabetes 52: 812–817. 12606524 10.2337/diabetes.52.3.812

[4] Larsen CM, Faulenbach M, Vaag A, Volund A, Ehses JA, et al (2007) Interleukin-1-receptor antagonist in type 2 diabetes mellitus. N Engl J Med 356: 1517–1526. 17429083 10.1056/NEJMoa065213

[5] Vandanmagsar B, Youm YH, Ravussin A, Galgani JE, Stadler K, et al (2011) The NLRP3 inflammasome instigates obesity-induced inflammation and insulin resistance. Nat Med 17: 179–188. 21217695 10.1038/nm.2279 PMC3076025

[6] Wen H, Gris D, Lei Y, Jha S, Zhang L, et al (2011) Fatty acid-induced NLRP3-ASC inflammasome activation interferes with insulin signaling. Nat Immunol 12: 408–415. 21478880 10.1038/ni.2022 PMC4090391

[7] Stienstra R, van Diepen JA, Tack CJ, Zaki MH, van de Veerdonk FL, et al (2011) Inflammasome is a central player in the induction of obesity and insulin resistance. Proc Natl Acad Sci U S A 108: 15324–15329. 21876127 10.1073/pnas.1100255108 PMC3174591

[8] Stienstra R, Mandard S, Tan NS, Wahli W, Trautwein C, et al (2007) The Interleukin-1 receptor antagonist is a direct target gene of PPARalpha in liver. J Hepatol 46: 869–877. 17321000 10.1016/j.jhep.2006.11.019

[9] McGillicuddy FC, Harford KA, Reynolds CM, Oliver E, Claessens M, et al (2011) Lack of interleukin-1 receptor I (IL-1RI) protects mice from high-fat diet-induced adipose tissue inflammation coincident with improved glucose homeostasis. Diabetes 60: 1688–1698. 21515850 10.2337/db10-1278 PMC3114387

[10] Stienstra R, Joosten LA, Koenen T, van Tits B, van Diepen JA, et al (2010) The inflammasome-mediated caspase-1 activation controls adipocyte differentiation and insulin sensitivity. Cell Metab 12: 593–605. 21109192 10.1016/j.cmet.2010.11.011 PMC3683568

[11] Kamari Y, Shaish A, Vax E, Shemesh S, Kandel-Kfir M, et al (2011) Lack of interleukin-1alpha or interleukin-1beta inhibits transformation of steatosis to steatohepatitis and liver fibrosis in hypercholesterolemic mice. J Hepatol 55: 1086–1094. 21354232 10.1016/j.jhep.2011.01.048 PMC3210940

[12] Harman-Boehm I, Bluher M, Redel H, Sion-Vardy N, Ovadia S, et al (2007) Macrophage infiltration into omental versus subcutaneous fat across different populations: effect of regional adiposity and the comorbidities of obesity. J Clin Endocrinol Metab 92: 2240–2247. 17374712 10.1210/jc.2006-1811

[13] Bergman RN, Kim SP, Hsu IR, Catalano KJ, Chiu JD, et al.. (2007) Abdominal obesity: role in the pathophysiology of metabolic disease and cardiovascular risk. Am J Med 120: S3–8; discussion S29–32. 10.1016/j.amjmed.2006.11.012 17296343

[14] Kabir M, Catalano KJ, Ananthnarayan S, Kim SP, Van Citters GW, et al (2005) Molecular evidence supporting the portal theory: a causative link between visceral adiposity and hepatic insulin resistance. Am J Physiol Endocrinol Metab 288: E454–461. 15522994 10.1152/ajpendo.00203.2004

[15] Sabio G, Das M, Mora A, Zhang Z, Jun JY, et al (2008) A stress signaling pathway in adipose tissue regulates hepatic insulin resistance. Science 322: 1539–1543. 19056984 10.1126/science.1160794 PMC2643026

[16] Wueest S, Rapold RA, Schumann DM, Rytka JM, Schildknecht A, et al (2010) Deletion of Fas in adipocytes relieves adipose tissue inflammation and hepatic manifestations of obesity in mice. J Clin Invest 120: 191–202. 19955656 10.1172/JCI38388 PMC2798678

[17] Rytka JM, Wueest S, Schoenle EJ, Konrad D (2011) The portal theory supported by venous drainage-selective fat transplantation. Diabetes 60: 56–63. 20956499 10.2337/db10-0697 PMC3012197

[18] Konrad D, Rudich A, Schoenle EJ (2007) Improved glucose tolerance in mice receiving intraperitoneal transplantation of normal fat tissue. Diabetologia 50: 833–839. 17334653 10.1007/s00125-007-0596-1

[19] Shaul ME, Bennett G, Strissel KJ, Greenberg AS, Obin MS (2010) Dynamic, M2-like remodeling phenotypes of CD11c+ adipose tissue macrophages during high-fat diet–induced obesity in mice. Diabetes 59: 1171–1181. 20185806 10.2337/db09-1402 PMC2857897

[20] Lagathu C, Yvan-Charvet L, Bastard JP, Maachi M, Quignard-Boulange A, et al (2006) Long-term treatment with interleukin-1beta induces insulin resistance in murine and human adipocytes. Diabetologia 49: 2162–2173. 16865359 10.1007/s00125-006-0335-z

[21] Jager J, Gremeaux T, Cormont M, Le Marchand-Brustel Y, Tanti JF (2007) Interleukin-1beta-induced insulin resistance in adipocytes through down-regulation of insulin receptor substrate-1 expression. Endocrinology 148: 241–251. 17038556 10.1210/en.2006-0692 PMC1971114

[22] Matsuki T, Horai R, Sudo K, Iwakura Y (2003) IL-1 plays an important role in lipid metabolism by regulating insulin levels under physiological conditions. J Exp Med 198: 877–888. 12975454 10.1084/jem.20030299 PMC2194201

[23] Suzawa M, Takada I, Yanagisawa J, Ohtake F, Ogawa S, et al (2003) Cytokines suppress adipogenesis and PPAR-gamma function through the TAK1/TAB1/NIK cascade. Nat Cell Biol 5: 224–230. 12598905 10.1038/ncb942

[24] Cawood TJ, Moriarty P, O'Farrelly C, O'Shea D (2006) The effects of tumour necrosis factor-alpha and interleukin1 on an in vitro model of thyroid-associated ophthalmopathy; contrasting effects on adipogenesis. Eur J Endocrinol 155: 395–403. 16914593 10.1530/eje.1.02242

[25] Nov O, Kohl A, Lewis EC, Bashan N, Dvir I, et al (2010) Interleukin-1beta may mediate insulin resistance in liver-derived cells in response to adipocyte inflammation. Endocrinology 151: 4247–4256. 20660063 10.1210/en.2010-0340

[26] Xu H, Barnes GT, Yang Q, Tan G, Yang D, et al (2003) Chronic inflammation in fat plays a crucial role in the development of obesity-related insulin resistance. J Clin Invest 112: 1821–1830. 14679177 10.1172/JCI19451 PMC296998

[27] Kosteli A, Sugaru E, Haemmerle G, Martin JF, Lei J, et al (2010) Weight loss and lipolysis promote a dynamic immune response in murine adipose tissue. J Clin Invest 120: 3466–3479. 20877011 10.1172/JCI42845 PMC2947229

[28] Persson J, Nilsson J, Lindholm MW (2008) Interleukin-1beta and tumour necrosis factor-alpha impede neutral lipid turnover in macrophage-derived foam cells. BMC Immunol 9: 70. 19032770 10.1186/1471-2172-9-70 PMC2596083

[29] Mosser DM, Edwards JP (2008) Exploring the full spectrum of macrophage activation. Nat Rev Immunol 8: 958–969. 19029990 10.1038/nri2448 PMC2724991

[30] Shaul ME, Bennett G, Strissel KJ, Greenberg AS, Obin MS (2010) Dynamic, M2-like Remodeling Phenotypes of CD11c+ Adipose Tissue Macrophages During High Fat Diet-Induced Obesity in Mice. Diabetes 59: 1171–1181. 20185806 10.2337/db09-1402 PMC2857897

[31] Zeyda M, Farmer D, Todoric J, Aszmann O, Speiser M, et al (2007) Human adipose tissue macrophages are of an anti-inflammatory phenotype but capable of excessive pro-inflammatory mediator production. Int J Obes (Lond) 31: 1420–1428. 17593905 10.1038/sj.ijo.0803632

[32] Guilherme A, Virbasius JV, Puri V, Czech MP (2008) Adipocyte dysfunctions linking obesity to insulin resistance and type 2 diabetes. Nat Rev Mol Cell Biol 9: 367–377. 18401346 10.1038/nrm2391 PMC2886982

[33] Fujiki K, Kano F, Shiota K, Murata M (2009) Expression of the peroxisome proliferator activated receptor gamma gene is repressed by DNA methylation in visceral adipose tissue of mouse models of diabetes. BMC Biol 9: 70. 10.1186/1741-7007-7-38 PMC2715379 19589179

[34] Sniderman AD, Bhopal R, Prabhakaran D, Sarrafzadegan N, Tchernof A (2007) Why might South Asians be so susceptible to central obesity and its atherogenic consequences? The adipose tissue overflow hypothesis. Int J Epidemiol 36: 220–225. 17510078 10.1093/ije/dyl245

[35] Kim JY, van de Wall E, Laplante M, Azzara A, Trujillo ME, et al (2007) Obesity-associated improvements in metabolic profile through expansion of adipose tissue. J Clin Invest 117: 2621–2637. 17717599 10.1172/JCI31021 PMC1950456

[36] Trak-Smayra V, Paradis V, Massart J, Nasser S, Jebara V, et al (2011) Pathology of the liver in obese and diabetic ob/ob and db/db mice fed a standard or high-calorie diet. Int J Exp Pathol 92: 413–421. 22118645 10.1111/j.1365-2613.2011.00793.x PMC3248077

[37] Persson H, Wallmark H, Ljungars A, Hallborn J, Ohlin M (2008) In vitro evolution of an antibody fragment population to find high-affinity hapten binders. Protein Eng Des Sel 21: 485–493. 18480091 10.1093/protein/gzn024

[38] Duewell P, Kono H, Rayner KJ, Sirois CM, Vladimer G, et al (2010) NLRP3 inflammasomes are required for atherogenesis and activated by cholesterol crystals. Nature 464: 1357–1361. 20428172 10.1038/nature08938 PMC2946640

[39] Rider P, Carmi Y, Guttman O, Braiman A, Cohen I, et al (2011) IL-1alpha and IL-1beta recruit different myeloid cells and promote different stages of sterile inflammation. J Immunol 187: 4835–4843. 21930960 10.4049/jimmunol.1102048

[40] Carmi Y, Rinott G, Dotan S, Elkabets M, Rider P, et al (2011) Microenvironment-derived IL-1 and IL-17 interact in the control of lung metastasis. J Immunol 186: 3462–3471. 21300825 10.4049/jimmunol.1002901

[41] Horai R, Asano M, Sudo K, Kanuka H, Suzuki M, et al (1998) Production of mice deficient in genes for interleukin (IL)-1alpha, IL-1beta, IL-1alpha/beta, and IL-1 receptor antagonist shows that IL-1beta is crucial in turpentine-induced fever development and glucocorticoid secretion. J Exp Med 187: 1463–1475. 9565638 10.1084/jem.187.9.1463 PMC2212263

[42] Ovadia H, Haim Y, Nov O, Almog O, Kovsan J, et al (2011) Increased adipocyte S-nitrosylation targets anti-lipolytic action of insulin: relevance to adipose tissue dysfunction in obesity. J Biol Chem 286: 30433–30443. 21724851 10.1074/jbc.M111.235945 PMC3162403

[43] Berry MN, Friend DS (1969) High-yield preparation of isolated rat liver parenchymal cells: a biochemical and fine structural study. J Cell Biol 43: 506–520. 4900611 10.1083/jcb.43.3.506 PMC2107801

[44] Bloch-Damti A, Potashnik R, Gual P, Le Marchand-Brustel Y, Tanti JF, et al (2006) Differential effects of IRS1 phosphorylated on Ser307 or Ser632 in the induction of insulin resistance by oxidative stress. Diabetologia 49: 2463–2473. 16896943 10.1007/s00125-006-0349-6

[45] Elgazar-Carmon V, Rudich A, Hadad N, Levy R (2008) Neutrophils transiently infiltrate intra-abdominal fat early in the course of high-fat feeding. J Lipid Res 49: 1894–1903. 18503031 10.1194/jlr.M800132-JLR200

[46] Cinti S, Mitchell G, Barbatelli G, Murano I, Ceresi E, et al (2005) Adipocyte death defines macrophage localization and function in adipose tissue of obese mice and humans. J Lipid Res 46: 2347–2355. 16150820 10.1194/jlr.M500294-JLR200

[47] Hao HX, Cardon CM, Swiatek W, Cooksey RC, Smith TL, et al (2007) PAS kinase is required for normal cellular energy balance. Proc Natl Acad Sci U S A 104: 15466–15471. 17878307 10.1073/pnas.0705407104 PMC2000499

