# Supplementary figures and images for: Interleukin-1β Regulates Fat-Liver Crosstalk in Obesity by Auto-Paracrine Modulation of Adipose Tissue Inflammation and Expandability
Source: PLoS One. 2013 Jan 16;8(1):e53626. doi: 10.1371/journal.pone.0053626 (PMC3547030; doi:10.1371/journal.pone.0053626)

## Slide 1
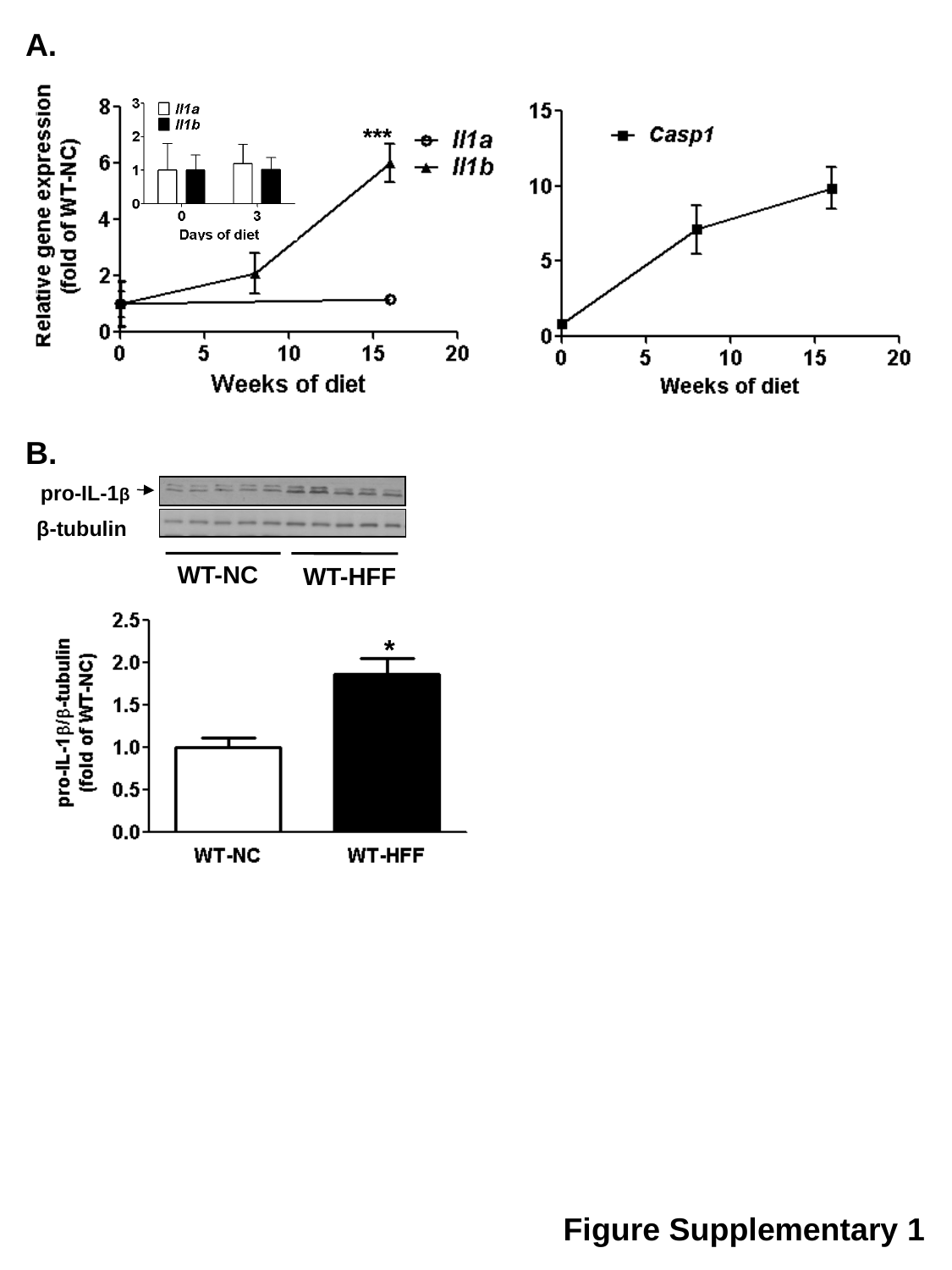

A.
***
***
**
B.
pro-IL-1β
β-tubulin
WT-NC
WT-HFF
*
Figure Supplementary 1

Supplement: Figure S1 — Increased adipose tissue expression of IL-1β in diet induced obesity. (A) Quantitative real-time PCR analysis of interleukin 1β (IL-1b), interleukin 1α (IL-1a) and caspase1 (casp1) in epididymal adipose tissue of C57/Bl6 wild-type (WT) mice during high fat feeding (HFF). Values are adjusted to 18S rRNA, and presented relative to age-matched littermates on normal chow diet (WT-NC). n = 5–12 per group/time-point; * p<0.05 compared to WT-NC at the same time point. (B) Representative western blot and densitometry analysis of pro-IL-1β in epididymal fat of 16 weeks high fat fed (HFF) or normal chow (NC) wild-type (WT) mice. A value of one was assigned to the mean pro-IL-1β to β-tubulin ratio in WT-NC. n = 5 in each group; *p<0.05. (PPT) [file pone.0053626.s001.ppt]

## Slide 1
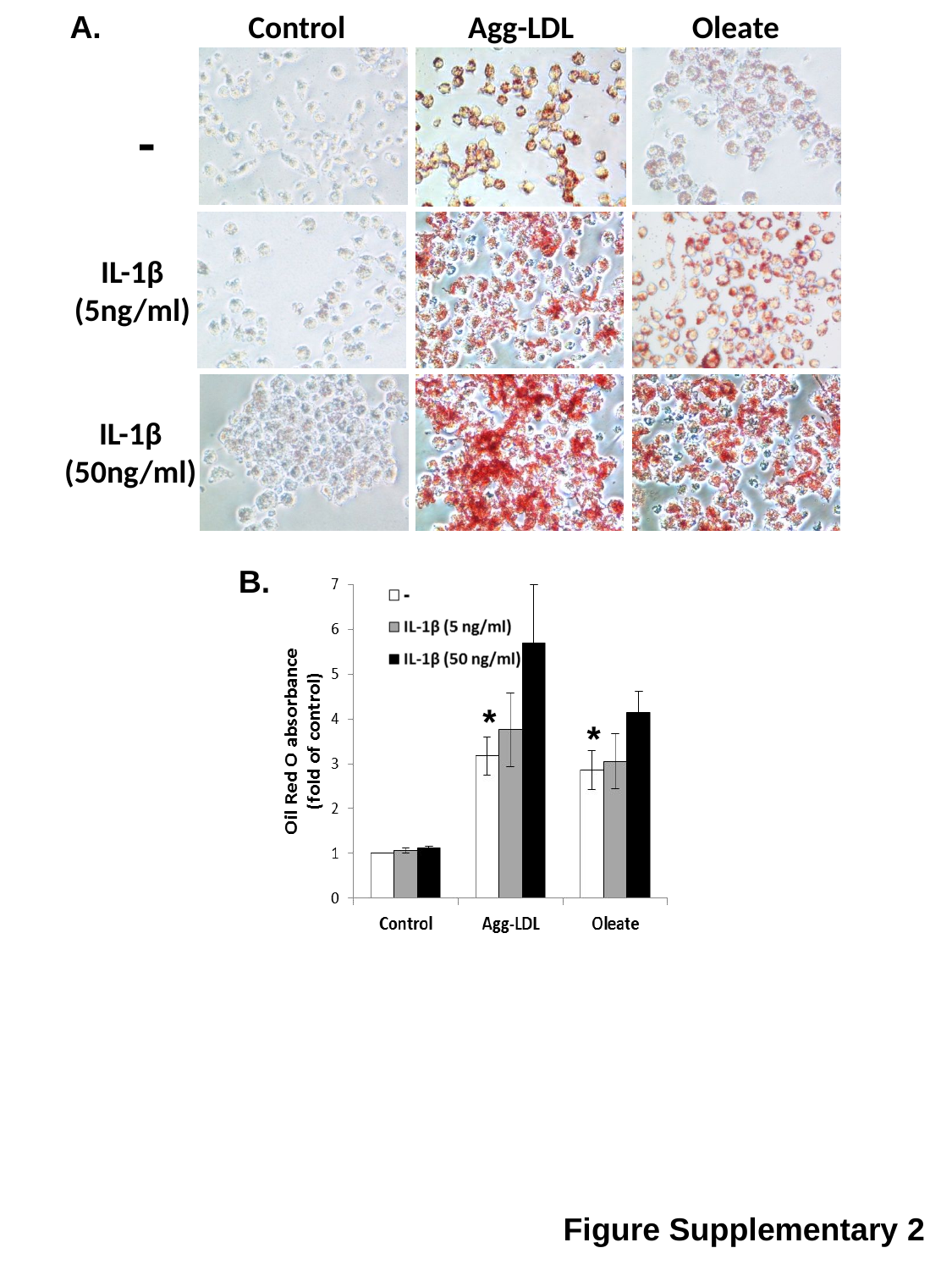

A.
Control
Agg-LDL
Oleate
-
IL-1β (5ng/ml)
IL-1β (50ng/ml)
B.
*
*
Figure Supplementary 2

Supplement: Figure S2 — IL-1β contributes to macrophage lipid accumulation. (A) J774.1 mouse macrophage cell line was incubated with aggregated LDL or with 0.5 mM oleic acid in the absence or presence of the indicated IL-1β concentrations. After 18 h cells were stained with oil red o to stain neutral lipids, and light microscopy images were taken. (B) Cells from 5 independent experiments were dissolved with DMSO and absorbance was determined using a microplate reader assigning an arbitrary value of 1 to control cells incubated in the absence of either lipids or IL-1. * p<0.05 compared to control. (PPT) [file pone.0053626.s002.ppt]

## Slide 1
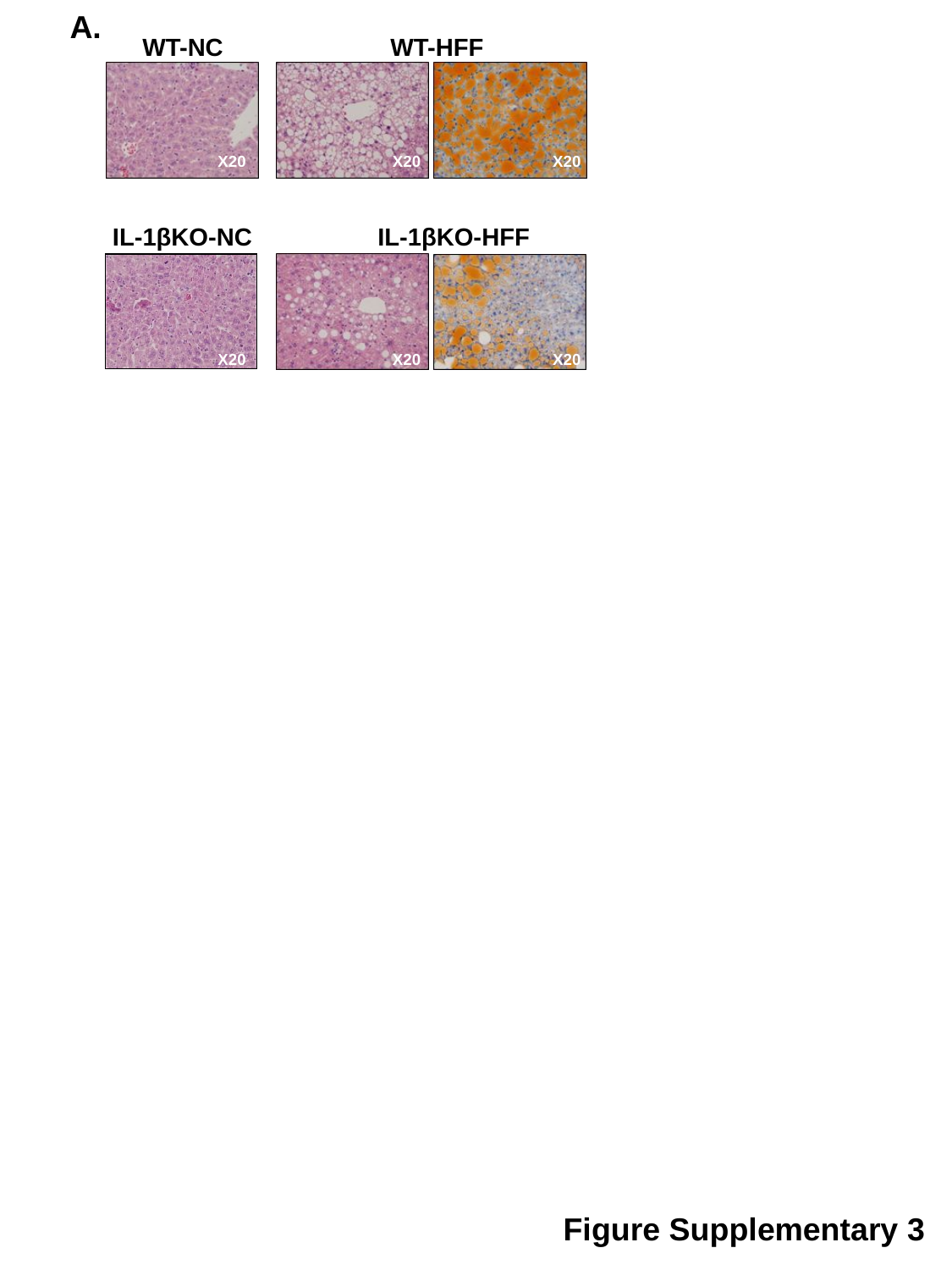

A.
 WT-NC WT-HFF
X20
X20
X20
 IL-1βKO-NC IL-1βKO-HFF
X20
X20
X20
Figure Supplementary 3

Supplement: Figure S3 — High fat diet induced hepatic steatosis is enhanced by IL-1β. Histological sections of livers of WT-NC, WT-HFF, IL-1βKO-NC and IL-1βKO-HFF stained for haematoxylin and eosin (H&E). WT-HFF and IL-1βKO-HFF were also stained for the neutral lipid stain Oil red O as detailed in methods. Shown are representative images of X20 light microscopy fields for H&E and oil red O. (PPT) [file pone.0053626.s003.ppt]

## Slide 1
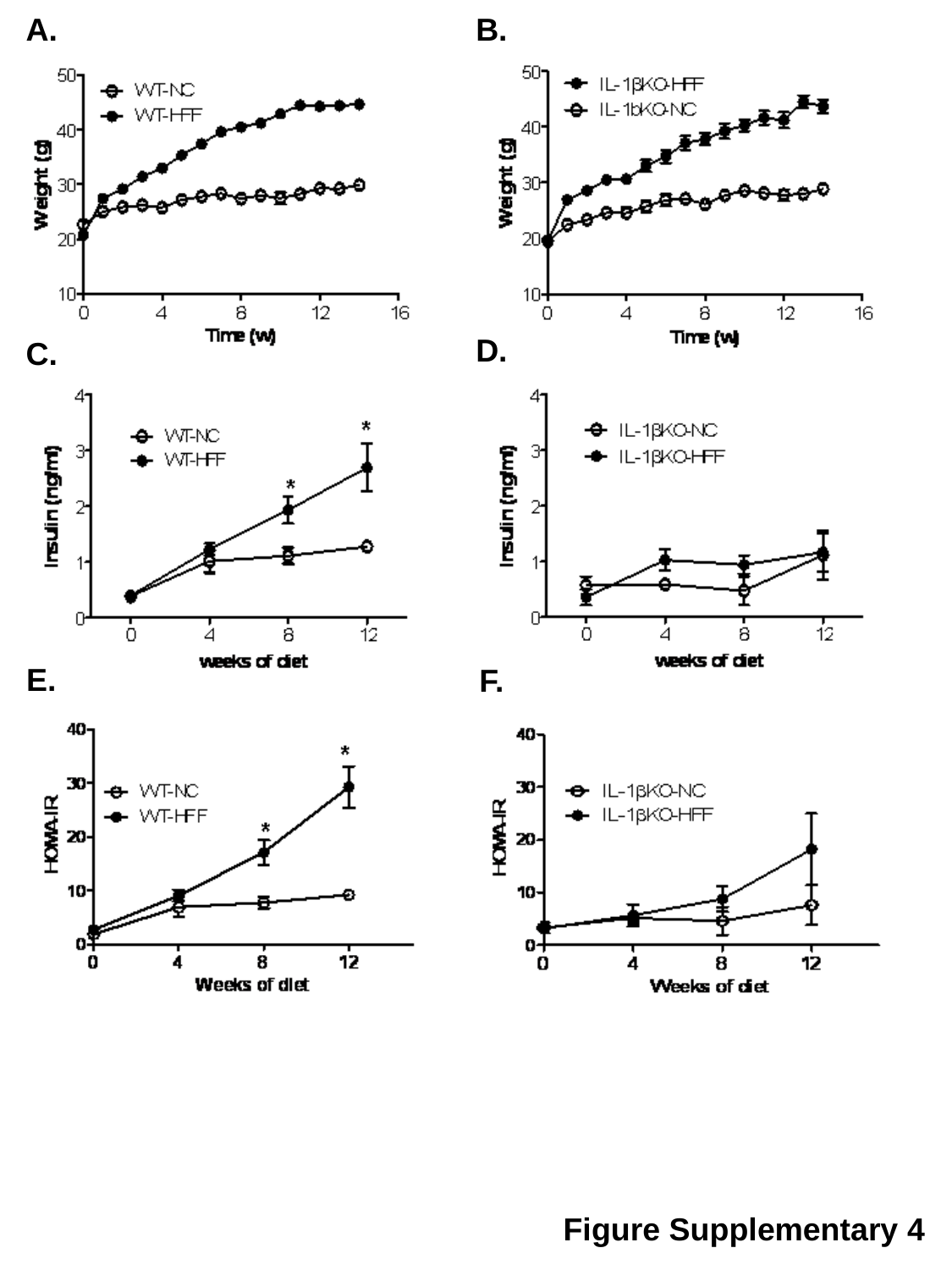

B.
A.
D.
C.
E.
F.
Figure Supplementary 4

Supplement: Figure S4 — IL-1β contributes to the development of fasting hyperinsulinemia in response to high-fat diet. (A–B) Body weight dynamics in WT-NC, WT-HFF (n = 8–16) and in IL-1β knockout mice on normal chow (IL-1βKO-NC) or high fat diet (IL-1βKO-HFF) (n = 6–13) as a function of weeks of dietary intervention. WT-HFF were not significantly different from IL-1βKO-HFF in any of the time points, but differed significantly (p<0.01) from WT-NC or from IL-1βKO-NC from week 3 respectively. The same groups were assessed for fasting insulin (C–D) and homeostasis model assessment insulin resistance (HOMA-IR)(E–F). * p<0.05 compared to WT-NC. (PPT) [file pone.0053626.s004.ppt]

## Slide 1
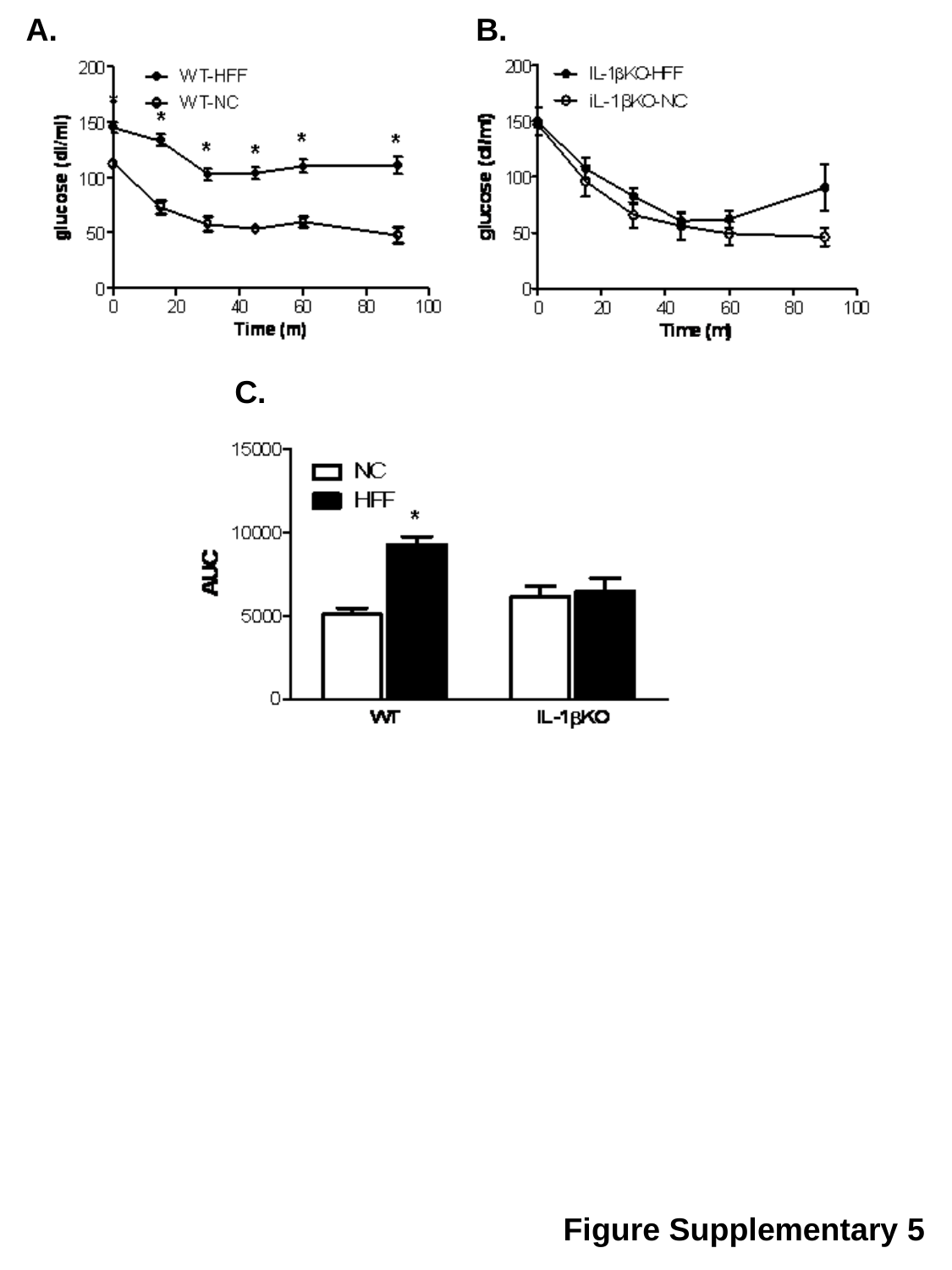

B.
A.
C.
Figure Supplementary 5

Supplement: Figure S5 — IL-1β contributes to whole-body insulin resistance in response to diet induced obesity. (A–B) Insulin tolerance test (ITT, 0.2 U/Kg body weight after 3 h fasting) after 12 weeks of HFF or normal chow, and (C) calculated area under the curve (AUC). * p<0.05 compared to WT-NC. (PPT) [file pone.0053626.s005.ppt]
