# Supplementary material for: Interleukin-1β Regulates Fat-Liver Crosstalk in Obesity by Auto-Paracrine Modulation of Adipose Tissue Inflammation and Expandability
Source: PLoS One. 2013 Jan 16;8(1):e53626. doi: 10.1371/journal.pone.0053626 (PMC3547030; doi:10.1371/journal.pone.0053626)
Supplement: Table S1 — List of Taq-man primers for quantitative real-time PCR. (PPT) [file pone.0053626.s006.ppt]

## Slide 1
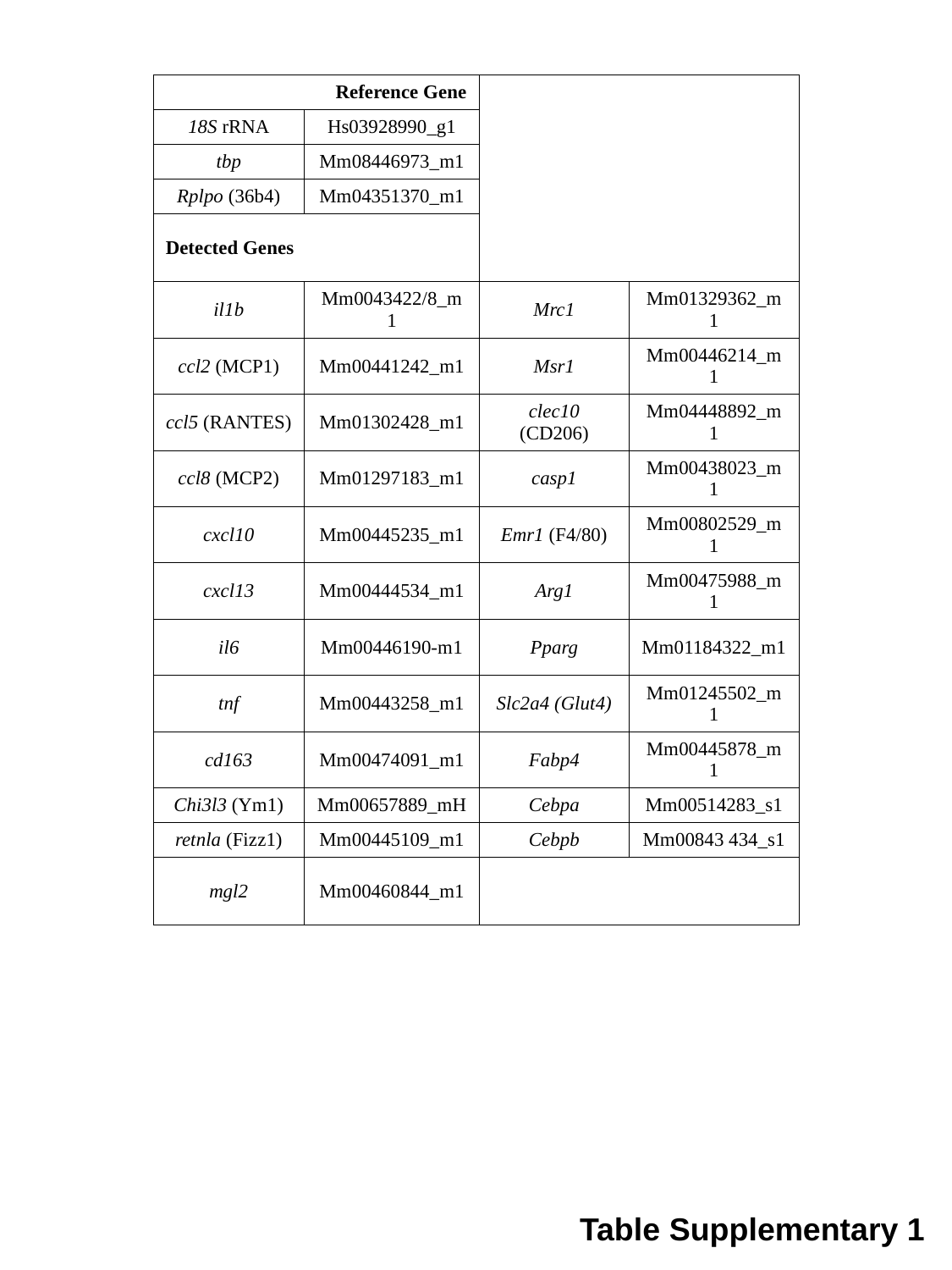

| Reference Gene | | | |
| --- | --- | --- | --- |
| 18S rRNA | Hs03928990\_g1 | | |
| tbp | Mm08446973\_m1 | | |
| Rplpo (36b4) | Mm04351370\_m1 | | |
| Detected Genes | | | |
| il1b | Mm0043422/8\_m1 | Mrc1 | Mm01329362\_m1 |
| ccl2 (MCP1) | Mm00441242\_m1 | Msr1 | Mm00446214\_m1 |
| ccl5 (RANTES) | Mm01302428\_m1 | clec10 (CD206) | Mm04448892\_m1 |
| ccl8 (MCP2) | Mm01297183\_m1 | casp1 | Mm00438023\_m1 |
| cxcl10 | Mm00445235\_m1 | Emr1 (F4/80) | Mm00802529\_m1 |
| cxcl13 | Mm00444534\_m1 | Arg1 | Mm00475988\_m1 |
| il6 | Mm00446190-m1 | Pparg | Mm01184322\_m1 |
| tnf | Mm00443258\_m1 | Slc2a4 (Glut4) | Mm01245502\_m1 |
| cd163 | Mm00474091\_m1 | Fabp4 | Mm00445878\_m1 |
| Chi3l3 (Ym1) | Mm00657889\_mH | Cebpa | Mm00514283\_s1 |
| retnla (Fizz1) | Mm00445109\_m1 | Cebpb | Mm00843 434\_s1 |
| mgl2 | Mm00460844\_m1 | | |
Table Supplementary 1
